# Feasibility of totally laparoscopic pylorus-preserving gastrectomy with intracorporeal gastro-gastrostomy for early gastric cancer: a retrospective cohort study

**DOI:** 10.1186/s12957-020-01955-z

**Published:** 2020-07-16

**Authors:** Yuji Akiyama, Akira Sasaki, Takeshi Iwaya, Ryosuke Fujisawa, Noriyuki Sasaki, Haruka Nikai, Fumitaka Endo, Shigeaki Baba, Yasushi Hasegawa, Toshimoto Kimura, Takeshi Takahara, Hiroyuki Nitta, Koki Otsuka, Keisuke Koeda

**Affiliations:** 1 Department of Surgery, Iwate Medical University School of Medicine, 2-1-1 Idaidori, Yahaba-cho, Shiwa-gun, Iwate, 028-3695 Japan; 2 Department of Medical Safety Science, Iwate Medical University School of Medicine, Iwate, Japan

**Keywords:** Totally laparoscopic pylorus-preserving gastrectomy, Gastric cancer, Intracorporeal reconstruction, Gastro-gastrostomy

## Abstract

**Background:**

Pylorus-preserving gastrectomy (PPG) has been accepted as a function-preserving surgery for the treatment of early gastric cancer in East Asian countries. Therefore, this study aimed to evaluate the feasibility and safety of totally laparoscopic PPG (TLPPG) with intracorporeal anastomosis.

**Methods:**

A total of 43 patients with early gastric cancer underwent laparoscopy-assisted PPG (LAPPG) with extracorporeal anastomosis between May 2006 and November 2012. The operative outcomes of 22 patients who underwent TLPPG between November 2012 and February 2019 were evaluated, and data were compared with that of the LAPPG group.

**Results:**

No significant difference in the operative time was observed between the two groups. Blood loss was lower in the TLPPG group (18.5 mL) than in the LAPPG group (30.7 mL, *p* = 0.008), and the length of abdominal incision was shorter in the TLPPG group (3.8 cm) than in the LAPPG group (4.7 cm, *p* < 0.001). No significant difference in the complication rate was observed between the two groups (13.6% in the TLPPG vs. 9.3% in the LAPPG group, *p* = 0.594). No anastomosis-related complications occurred in either group. No significant between-group difference was observed in the delayed gastric emptying (TLPPG, 9.1 vs. LAPPG, 7%, *p* = 0.762). The initiation of postoperative fluid (TLPPG, 1.0 day vs. LAPPG, 3.0 days, *p* < 0.001) and meal (TLPPG, 3.0 days vs. LAPPG, 4.0 days, *p* < 0.001) intake was earlier in the TLPPG group than in the LAPPG group. No significant between-group difference was observed in the postoperative hospital stay.

**Conclusions:**

The findings of this study suggest that TLPPG with intracorporeal reconstruction not only is as feasible and safe as LAPPG for the treatment of patients with early gastric cancer but also provides certain advantages such as reduced blood loss and wound size.

## Background

Pylorus-preserving gastrectomy (PPG) has been accepted as a function-preserving surgery for the treatment of early gastric cancer in East Asian countries [1–4]. Compared with distal gastrectomy, PPG has many advantages in terms of preventing postoperative disorders such as dumping syndrome, bile reflux, and gallstone formation [2–4]. According to the Japanese Gastric Cancer Treatment guidelines, PPG is recommended as an optional method for early gastric cancer located in the middle third of the stomach [5]. In recent years, laparoscopy-assisted PPG (LAPPG) has been increasingly demonstrated [6, 7]. The safety of LAPPG with a hand-sewn gastro-gastrostomy performed under a mini-laparotomy has been reported [6, 7]. Moreover, totally laparoscopic PPG (TLPPG), in which the anastomosis was performed intracorporeally, has also been reported [8–10].

With regard to the laparoscopic distal gastrectomy, several studies have demonstrated the usefulness of minimally invasive totally laparoscopic distal gastrectomy (TLDG) with intracorporeal anastomosis and compared this procedure with laparoscopy-assisted distal gastrectomy (LADG) with extracorporeal anastomosis under mini-laparotomy [11–13]. Compared with LADG, TLDG has been shown to reduce wound size and blood loss, fasten recovery time, and shorten the postoperative hospital stay [11–13]. The operative procedure of PPG was changed from LAPPG to TLPPG in November 2012. In this study, the relative operative outcomes of LAPPG and TLPPG were investigated, and the feasibility and safety of TLPPG were evaluated.

## Methods

### Patients

A total of 66 consecutive patients with early gastric cancer who underwent laparoscopic PPG between May 2006 and February 2019 were retrospectively reviewed. Among these, 43 underwent LAPPG between May 2006 and November 2012, and 23 underwent TLPPG between November 2012 and February 2019. The first case of TLPPG was performed via intracorporeal totally hand-sewn anastomosis. From the second case onward, a hybrid anastomotic procedure was devised using linear staplers on the posterior wall and hand-sewn anastomosis on the anterior wall of the stomach, and the procedure was standardized using this method [9]. Therefore, we analyzed 22 patients in the TLPPG group, excluding the first one, in this study. LAPPG and TLPPG were indicated for clinical stage I (T1N0M0) gastric cancer in the middle third of the stomach, according to the Japanese Gastric Cancer Treatment guidelines (version 5) [5]. Clinical and pathological stages were classified according to the Japanese Classification of Gastric Carcinoma [5].

### Surgical procedure

In this study, laparoscopic PPG was performed by two surgeons in the same group. Surgical procedures for laparoscopic PPG were described in our previous study [9]. In principle, the infra-pyloric artery and celiac branch of the vagus nerve were preserved. The procedure was modified in 2012 to promote preservation of the infra-pyloric vein (IPV).

In TLPPG, the resected specimen was obtained via the 3–4 cm incision at the opened original umbilical port site in gastrectomy. Reconstruction was performed intracorporeally with an end-to-end gastro-gastrostomy. Anastomosis was performed using a linear stapler on the posterior wall and layer-to-layer hand-sewn anastomosis on the anterior wall [9]. In LAPPG, although gastrectomy was performed using the same method as TLPPG, the reconstruction procedure was performed via a 4–5 cm incision at the upper middle or right hypochondrium transverse regions. Anastomosis was performed extracorporeally via a continuous layer-to-layer hand-sewn anastomosis.

In our clinical pathway, between May 2006 and September 2010, postoperative fluid intake and meal were started on postoperative days (POD) 3 and 4, respectively. However, since October 2010, fluid intake and meal were initiated earlier on POD 1 and 3, respectively. Discharge was set at POD 10 in both groups.

### Postoperative complications

Postoperative complications were defined according to the Clavien–Dindo classification [14]. Delayed gastric emptying (DGE), including gastric stasis, was also defined according to the Clavien–Dindo classification and diagnosed according to Kaji et al.’s definition, i.e., upper abdominal distension, nausea, or vomiting, accompanied with the retention of the remnant stomach on abdominal X-ray [15].

### Statistical analysis

Statistical analyses were performed using the SAS statistical analysis software, JMP 10 (SAS, Cary, NC, USA). Differences in patient characteristics and outcomes between the two groups were estimated using the *χ*^2^, Student’s, or Wilcoxon’s rank tests. A *p* < 0.05 was considered statistically significant.

## Results

### Patient characteristics

Patient characteristics are shown in Table 1. No significant differences were observed in patient characteristics between the two groups. In the LAPPG group, one patient had a clinical T2 tumor. The tumor was resected with sufficient surgical margin in this patient, and PPG was performed after obtaining informed consent.

**Table 1:** Patient characteristics

	TLPPG, *n* = 22	LAPPG, *n* = 43	*p* value
Age (years)^a^	57.8 ± 10.5	60.4 ± 12.7	0.409
Sex			0.804
Male	8	17	
Female	14	26	
BMI (kg/m^2^)^a^	21.3 ± 1.8	22.4 ± 3.2	0.064
ASA score			0.090
1	11	11	
2	11	29	
3	0	3	
Clinical T stage^b^			0.700
T1a	8	13	
T1b	14	29	
T2	0	1	
Pathological T stage^b^			0.814
T1a	10	22	
T1b	10	15	
T2	1	2	
T3	1	4	
Pathological N stage^b^			0.171
N0	17	40	
N1	4	2	
N2	1	1	
Pathological stage^b^			0.331
IA	15	34	
IB	5	4	
IIA	2	5	
History of abdominal operation			0.503
Yes	4	11	
No	18	32	
ESD preoperatively			0.638
Yes	4	10	
No	18	33	

*BMI* body mass index, *ASA* American Society of Anesthesiologists, *ESD* endoscopic submucosal resection

^a^Mean ± standard deviation

^b^According to the Japanese Classification of Gastric Carcinoma, 15th edition

### Surgical and postoperative outcomes

Surgical procedures and operative outcomes are presented in Table 2. No significant difference in the operative time was observed between the two groups. Blood loss was lower in the TLPPG group (18.5 mL) than in the LAPPG group (30.7 mL, *p* = 0.008), and length of abdominal incision was shorter in the TLPPG group (3.8 cm) than in the LAPPG group (4.7 cm, *p* < 0.001). All patients underwent complete resection. No intraoperative complication or conversion to open laparotomy occurred.

**Table 2:** Surgical procedures and operative outcomes

	TLPPG, *n* = 22	LAPPG, *n* = 43	*p* value
Operation time (min)^a^	264.3 ± 37.3	246.7 ± 49.1	0.145
Blood loss (mL)^a^	18.5 ± 13.7	30.7 ± 22.2	0.008
Lymph node dissection^b^			0.985
D1	1	2	
D1+	21	41	
Co-resection			0.247
Yes	1	6	
No	21	37	
Celiac branch of the vagus nerve			0.471
Preserved	22	42	
Not preserved	0	1	
Infra-pyloric artery			0.159
Preserved	21	43	
Not preserved	1	0	
Infra-pyloric vein			< 0.001
Preserved	22	5	
Not preserved	0	38	
Length of abdominal incision (cm)^a^	3.8 ± 0.4	4.7 ± 0.6	< 0.001
Number of the harvested lymph nodes^a^	39.5 ± 11.6	37.7 ± 14.8	0.635
Conversion to open laparotomy	0	0	–

^a^Mean ± standard deviation

^b^According to the Japanese Gastric Cancer Treatment Guideline 2018

Postoperative complications are shown in Table 3. No significant difference in the complication rate was observed between the two groups (TLPPG 13.6% vs. LAPPG 9.3%, *p* = 0.594). The complication severity in all patients was grade II according to the Clavien–Dindo classification. No significant between-group difference was observed in DGE (TLPPG 9.1 vs. LAPPG 7%, *p* = 0.762). No operative mortality occurred in either group.

**Table 3:** Postoperative complications

	TLPPG, *n* = 22 (%)	LAPPG, *n* = 43 (%)	*p* value
Morbidity^a^	3 (13.6)	4 (9.3)	0.594
Anastomotic leakage	0	0	–
Anastomotic bleeding	0	0	–
Anastomotic ulcer	0	0	–
Anastomotic stenosis	0	0	–
Pancreatic fistula	0	0	–
Intra-abdominal abscess	0	0	–
Delayed gastric emptying	2 (9.1)	3 (7)	0.762
Pneumonia	2 (9.1)	3 (7)	0.762
Wound infection	0	0	–
Ileus	0	0	–
30-day mortality	0	0	–

^a^There is some overlap

Postoperative outcomes are shown in Table 4. No significant differences were observed in body temperature at maximal values within POD 3, and white blood cell (WBC) counts and C-reactive protein (CRP) at maximal values occurred within POD 7. Postoperative fluid (TLPPG, 1.0 day vs. LAPPG, 3.0 days, *p* < 0.001) and meal (TLPPG, 3.0 days vs. LAPPG, 4.0 days, *p* < 0.001) intake were initiated earlier in the TLPPG group than that in the LAPPG group. No significant between-group difference was observed in postoperative hospital stay (Table 4). Although the site of incision was different between the two groups, there was no difference in patient management during the perioperative and follow-up periods.

**Table 4:** Postoperative outcomes

	TLPPG, *n* = 22	LAPPG, *n* = 43	*p* value
Body temperature^a,b^	37.8 ± 0.4	37.9 ± 0.6	0.526
Postoperative blood test^a^			
WBC (/μL)^c^	9688.2 ± 2567.8	9655.8 ± 3473.5	0.970
CRP (mg/dL)^c^	8.6 ± 5.3	7.1 ± 5.3	0.278
Amylase (U/L)^d^	263.9 ± 321.2	250.3 ± 301.9	0.867
Ambulation (days)^a,e^	1.0 ± 0.2	1.1 ± 0.3	0.705
Fluid intake (days)^e,f^	1.0 (1–2)	3.0 (1–5)	< 0.001
Meal intake (days)^e,f^	3.0 (3–5)	4.0 (3–5)	< 0.001
Postoperative hospital stay (days)^f^	10 (8–40)	10 (7–18)	0.2412

*CRP* C-reactive protein, *WBC* white blood cell

^a^Mean ± standard deviation

^b^Maximal values within POD 3

^c^Maximal values within POD 7

^d^Values at POD 1

^e^First-day postoperatively

^f^Median (range)

## Discussion

TLPPG is a surgical procedure performed laparoscopically. In particular, the intracorporeal anastomosis technique is more complicated than the LAPPG with extracorporeal anastomosis, and the gastric lumen is exposed in the abdominal cavity; therefore, an increase in anastomosis-related complications or intra-abdominal infection was predicted. The present study evaluated the feasibility and safety of TLPPG. No significant differences were observed in the operative time and complication rates between the LAPPG and TLPPG groups (Tables 2 and 3). These results suggested that TLPPG was as safe as LAPPG for patients with early gastric cancer in this study.

The operative blood loss was significantly lower in the TLPPG group than in the LAPPG group (Table 2). Several studies have reported that blood loss is significantly lower in TLDG patients than in LADG patients during distal gastrectomy examination [11, 16]. Although the influence of operative learning curve was confirmed by reduced blood loss because TLDG was performed during the latter period in this study [16], the difference in wound length of abdominal incision might affect the blood loss result. The number of wounds was smaller in TLPPG (5 sites) than in LAPPG (6 sites) because only a small incision was made to remove the resected specimen at the umbilical port site in TLPPG. In addition, the small incision was significantly shorter (3.8 cm) in TLPPG than in LAPPG (4.7 cm) (Table 2), which might also affect blood loss. Moreover, compared with extracorporeal anastomosis with restricted vision through mini-laparotomy, especially in obese patients or with a small remnant stomach, the intracorporeal anastomosis procedure performed in a sufficient laparoscopic view might be partly responsible for the reduced blood loss [11, 12]. The intra-abdominal pressure generated by the pneumoperitoneum in laparoscopy could help reduce bleeding during the anastomosis procedure in TLPPG [17].

The size of the small incision in LAPPG primarily depends on the thickness of the body wall of patients or the size of remnant stomach; however, in TLPPG, the size of the small incision can unify without depending on patient factors. This fact might be a strong point for TLPPG compared with LAPPG. Previous reports suggest that the postoperative inflammatory response is lower in TLDG patients than in LADG patients [11, 16]. Ikeda et al. reported that serum CRP levels on POD 7 were significantly lower in TLDG patients than in LADG patients [11]. Lee et al. also reported that postoperative WBC counts and serum CRP levels were significant lower in the TLDG group than in the LADG group [16]. Similarly, in the present study, we hypothesized that the inflammatory response would be lower in the TLPPG group than in the LAPPG group owing to the fact that TLPPG results in a reduced wound incision and consequently reduced tissue damage to the abdominal wall. However, no significant differences in postoperative WBC counts or serum CRP levels were observed between the two groups. The small difference in incisions length, approximately 1 cm in this study, might not be reflected in the postoperative inflammatory response. Postoperative fluid and meal intake were initiated earlier in the TLPPG group than in the LAPPG group (Table 4). These results may also have been influenced by the clinical pathway of enhanced recovery after surgery (ERAS). The ERAS program reduces the time required for normalization of gut function [18]. With the widespread use of the recent ERAS protocol, fluid and meal intakes were initiated earlier in the latter period of this study. Most of the patients in the TLPPG group started fluid intake on POD 1 and meal intake on POD 3. However, there was no difference between the two groups in the postoperative hospital stay because the discharge was set at POD 10 in both groups for the purpose of adequate observation of the postoperative meal intake.

DGE including gastric stasis is one of the most common problems after PPG [1, 6, 7]. Its incidence has been reported to be 6.3–10% in recent years [1, 6–8, 15, 19]. In our study, the incidence of DGE was 9.1% in the TLPPG group, but it did not decrease in the LAPPG group (7%). Preservation of IPV in addition to the infra-pyloric artery has reportedly been useful for the prevention of DGE [19]. Kiyokawa et al. demonstrated that venous stasis due to IPV division could cause pyloric edema, which might cause pyloric dysfunction and DGE [19]. However, Kaji et al. reported that preserving IPV did not help prevent DGE in laparoscopic and robotic PPG [15]. The influence of preserving IPV for DGE after PPG remains controversial. In our study, IPV was preserved in all patients in the TLPPG group (Table 2). No difference in the DGE incidence was observed between the TLPPG with IPV preservation and LAPPG without IPV preservation in the majority of patients.

Regarding postoperative remnant stomach function, a hand-sewn anastomosis may be more advantageous for remnant stomach deformation or anastomosis flexibility than staples [20]. Namikawa et al. studied quality of life of patients who underwent PPG and reported that nausea scores were significantly lower in patients who underwent hand-sewn anastomosis than in those who underwent anastomosis with a linear stapler [20]. Although hand-sewn anastomosis is preferred after PPG and totally hand-sewn anastomosis was only performed once intracorporeally during the TLPPG procedure, securing a sufficient lumen of anastomosis remains difficult due to contraction of the stomach wall [9]. A hybrid anastomotic procedure was therefore devised using linear staplers on the posterior wall and hand-sewn anastomosis on the anterior wall of the stomach [9]. No adverse events related to anastomosis occurred in the TLPPG group in this short-term study, but a long-term functional examination should be conducted in the future. We believe that our method of intracorporeal anastomosis is as safe as extracorporeal anastomosis.

The present study demonstrated that TLPPG is a feasible and safe procedure for the treatment of early gastric cancer. However, several limitations were observed in our study. First, this was a retrospective cohort study with a small study population in a single center. Second, selection bias and an operative learning curve were inevitable because patients underwent TLPPG during the latter period of this study. Lastly, we did not perform nutritional analysis or assess either quality of life or long-term outcomes of the patients. To confirm the usefulness of TLPPG, a well-designed large-scale prospective study is warranted.

## Conclusions

The findings of this study suggest that because of the advantages of reduced blood loss and wound size, TLPPG with intracorporeal reconstruction is as feasible and safe as LAPPG for the treatment of patients with early gastric cancer.

## Data Availability

The datasets used and/or analyzed during this study are available from the corresponding author on reasonable request.

## Abbreviations

CRP: C-reactive protein
DGE: Delayed gastric emptying
ERAS: Enhanced recovery after surgery
IPV: Infra-pyloric vein
LAPPG: Laparoscopy-assisted pylorus-preserving gastrectomy
POD: Postoperative day
TLPPG: Totally laparoscopic pylorus-preserving gastrectomy
WBC: White blood cell
